# Hypoglycemic activity of *Hemidesmus indicus* R. Br. on streptozotocin-induced diabetic rats

**DOI:** 10.4103/0973-3930.41979

**Published:** 2008

**Authors:** Mahalingam Gayathri, Krishnan Kannabiran

**Affiliations:** Biomolecules and Genetics Division, School of Biotechnology, Chemical and Biomedical Engineering, VIT University, Vellore, Tamil Nadu, India

**Keywords:** Antidiabetic activity, blood glucose, *Hemidesmus indicus*, lipid peroxides, streptozotocin

## Abstract

**OBJECTIVE:**

To evaluate the antidiabetic activity of an aqueous extract of the roots of *Hemidesmus indicus* on blood glucose, serum electrolytes, serum marker enzymes, liver microsomal P-450 enzymes, and lipid peroxidation in the liver and kidney of streptozotocin-induced diabetic rats.

**MATERIALS AND METHODS:**

Effect of *H. indicus* extract on blood glucose was studied with fed, fasted and glucose-loaded diabetic and nondiabetic rat models. The effect of the extract on serum electrolytes, serum levels of key glucose metabolizing enzymes, hepatic microsomal protein and hepatic cytochrome P-450-dependent mono-oxygenase enzyme systems and lipid peroxidation in the liver and kidney of diabetic rats. One way analysis of variance and Duncan's multiple range test was used for statistical analysis.

**RESULTS:**

Oral administration of *H. indicus* aqueous extract to fed, fasted and glucose-loaded diabetic rats decreased blood glucose level significantly at 5 h and restored serum electrolytes, glycolytic enzymes and hepatic cytochrome P-450-dependent enzyme systems by preventing the formation of liver and kidney lipid peroxides at the end of 12 weeks of the study period.

**CONCLUSION:**

From the studies, it can be concluded that the aqueous extract of the roots of *H. indicus* at a dosage of 500 mg/kg/day exhibits significant antidiabetic activity. It restores the concentrations of electrolytes, glucose metabolizing enzymes, hepatic microsomal protein and hepatic cytochrome P-450-dependent mono-oxygenase enzyme systems to near normal level and also corrects the related metabolic alterations in experimentally induced diabetic rats. *H. indicus* administration also decreased liver and kidney lipid peroxidation products. On the basis of our findings, *H. indicus* could be used as an antidiabetic and antioxidant agent for the prevention and treatment of diabetes mellitus.

## Introduction

In the treatment of diabetes mellitus, non-pharmacologic measures (e.g., diet, exercise, and weight loss) remain a critical component of therapy. Dietary management includes the use of traditional medicines that are mainly derived from plants.[1] Even now, approximately 80% of the third world population is almost entirely dependent on traditional medicines.[2] The ethnobotanical details and the antidiabetic potential of several plants are known at present,[3] but there is very little information about plants which possess both hypoglycemic and antioxidant properties; such a plant would be very useful as an antidiabetic agent.

*H. indicus* R. Br. has been reported to be used as a tonic, demulcent, and diaphoretic and has traditionally been used to treat venereal diseases, skin diseases, urinary infections, negative emotions and impotence.[3] It also prevents abdominal distention, arthritis, rheumatism, gout, and epilepsy.[4] The active principle of *H. indicus*, 2-hydroxy 4-methoxy benzoic acid (HMBA), was reported to be hepatoprotective in ethanol-induced hepatotoxicity in rats.[5] There is no report on the hypoglycemic potential of this plant extract so far. The main objective of this study was to assess the antidiabetic effect of an aqueous extract of the roots of *H. indicus* in controlling the blood glucose levels and its effectiveness on various other related metabolic biochemical parameters.

## Materials and Methods

Male albino rats (Wistar strain, weighing 150-200 g) were purchased from Tamil Nadu Veterinary Animal Science University, Madhawaram, Chennai, and housed under standard husbandry conditions (30°C ± 2°C, 60-70% relative humidity, and 12 h: 12 h day-night cycle) and allowed standard pellet rat feed and water *ad libitum*. The animal experiments were designed and conducted in accordance with the guidelines of the Institutional Animal Ethical Committee (IAEC), VIT University.

The roots of *H. indicus* were collected from the Morappur forest area, Dharmapuri district, Tamil Nadu, during the month of April 2005. The plants were authenticated by the Forest Department, Dharmapuri district, Tamil Nadu, where a voucher specimen (FDSC 201) was also submitted. The roots of *H. indicus* was washed with distilled water, shade dried, powdered, and stored in an air-tight container until further use.

The powder of the root of *H. indicus* (100 g) was mixed with 500 ml of sterile distilled water and a juice was obtained using a Turmix electric extractor. The juice was filtered and the residue was removed. The extract was concentrated under vacuum to obtain a solid, which was then freeze-dried (Thermo freeze-drier, USA); the yield was calculated.

Diabetes was induced experimentally in rats by a single intraperitoneal injection of a freshly prepared solution of streptozotocin (STZ) (Sigma, USA) at a dose of 35 mg/kg body weight (bw) in 0.1 M cold citrate buffer of pH 4.5. After 72 h, blood was collected with all aseptic precautions from the tail vein of the rats under ether anesthesia and blood glucose levels were determined using Autoanalyser Microlab 2000 (Hamilton, UK). The animals were considered to be diabetic if the blood glucose values were above 250 mg/dl, and those animals alone were used for the study. Diabetes was developed and stabilized in STZ-treated rats over a period of 7 days.[6] Control rats were given citrate buffer (pH 4.5). The hypoglycemic activity of *H. indicus* aqueous extract was assessed by feeding the test extract (500 mg/kg bw/day) to diabetic control, nondiabetic, and drug-treated diabetic animals (fasted, fed, and glucose-loaded models). The animals were followed up to 5 h to check the time required for the test extract to produce peak hypoglycemic activity.

The animals were divided into six groups of six animals each. Group I served as a control; group II had STZ-treated surviving diabetic rats; group III served as a positive control and received a standard hypoglycemic agent, tolbutamide (100 mg/kg bw/day); group IV comprised the fasted rat model: nondiabetic and diabetic rats were fasted for 12 h and treated with the aqueous extract, 500 mg/kg body weight/day, by oral intubation method. Group V comprised fed rats: nondiabetic and diabetic rats were provided with an unlimited quantity of rat feed and treated with aqueous extract, 500 mg/kg bw/day, by oral intubation method. Group VI had the glucose-loaded model: nondiabetic and diabetic rats were fasted for 12 h and treated with aqueous extract, 500 mg/kg bw/day, by oral intubation method, followed by 10% glucose solution (1.5 g/kg bw). The rats were followed for 5 h, with the blood sugar levels being estimated at the end of 1 h, 3 h and 5 h. Test drug treatment was continued for 12 weeks, at the end of which the rats were sacrificed. Blood samples and liver and kidney tissue were collected to carry out the biochemical studies.

Plasma glucose was estimated by the glucose oxidase method.[7] The serum was assayed for the electrolytes sodium, potassium and calcium. Serum marker enzymes and liver microsomal mono-oxygenase enzymes were estimated by using commercial kits purchased from Bayer Diagnostics India Ltd. Liver and kidney thiobarbituric acid reactive substances[8] and hydroperoxides[9] were estimated.

Statistical analysis was performed using the SPSS software package, version 9.05. The values were analyzed by one way analysis of variance (ANOVA) followed by Duncan's multiple range test (DMRT). All the results were expressed as mean ± SD for six rats in each group and *P* < 0.05 was considered significant.

## Results

The yield of aqueous extract of *H. indicus* roots was found to be 3.7% (w/v). STZ treatment increased blood glucose levels significantly (*F* > 0.05; *P* < 0.001) in experimental rats when compared to normal rats. Administration of *H. indicus* aqueous extract decreased blood glucose levels significantly (*F* > 0.05; *P* < 0.001) at 1 h, 3 h and 5 h in STZ-induced diabetic rats in the fasted, fed, and glucose-loaded models [Table 1].

**Table 1:** Effect of *H. indicus* on plasma glucose levels in normal and streptozotocin-induced diabetic rats

Groups	Experimental condition	Dose (mg/kg bw/day)	1 h (mg/dl)	3 h (mg/dl)	5 h (mg/dl)
Normal	-	35 ml/water	167 ± 2.09	167 ± 2.09	167 ± 2.09
Diabetic control	-	35 ml/water	378 ± 2.28	378 ± 2.28	378 ± 2.28
Diabetic + Tolbutamide	-	100	295 ± 2.98*	282 ± 2.96*	274 ± 2.52*
Diabetic + *H. indicus*	ND	500	165 ± 3.21	162 ± 1.24	158 ± 2.54
(fasted model)	D	500	270 ± 3.54*	269 ± 1.87*	265 ± 2.87*
Diabetic + *H. indicus*	ND	500	183 ± 3.60	169 ± 3.01	167 ± 2.84
(fed model)	D	500	297 ± 1.0*	286 ± 1.1*	276 ± 1.40*
Diabetic + *H. indicus*	ND	500	172 ± 1.02	170 ± 2.02	169 ± 1.87
(glucose-loaded model)	D	500	281 ± 1.63*	273 ± 2.35*	267 ± 2.35*

ND - nondiabetic rats; D - diabetic rats; Each value is mean ± SD for six rats in each group

* Values are statistically significant when compared to diabetic control at *F* > 0.05 (ANOVA) and *P* < 0.05 (DMRT)

The metabolic enzymes of glucose were significantly decreased (*F* > 0.05; *P* < 0.001) in STZ-induced diabetic rats when compared to normal control rats. Oral administration of the aqueous extract of *H. indicus* root had a significant effect (*F* > 0.05; *P* < 0.001) in restoring the levels of the metabolic enzymes to near-normal levels [Table 2].

**Table 2:** Effect of *H. indicus* extract on serum marker enzymes GS, GK, LDH, SD, and MD in normal and streptozotocin-induced diabetic rats

Groups	Dose (mg/kg bw/day)	GS (U/l)	GK (U/l)	LDH (U/l)	SD (U/l)	MD (U/l)
Normal	35 ml/water	8.07 ± 1.25	9.07 ± 2.52	92.74 ± 2.13	4.84 ± 125	5.85 ± 1.42
Diabetic control	35 ml/water	5.55 ± 1.36	4.12 ± 2.31	60.84 ± 1.54	3.14 ± 1.45	1.87 ± 1.32
Diabetic + tolbutamide	100	6.17 ± 1.58*	8.65 ± 1.95*	89.40 ± 1.45*	6.74 ± 1.28*	4.32 ± 1.62*
Diabetic + *H. indicus*	500	7.85 ± 2.01*	8.74 ± 2.63*	90.25 ± 1.24*	6.95 ± 1.68*	4.85 ± 2.03*

Each value is mean ± SD for six rats in each group.; GS - glycogen synthase; GK - glucokinase; LDH - lactate dehydrogenase; SD - succinate dehydrogenase; MD - malate dehydrogenase

* Values are statistically significantly when compared to diabetic control at *F* > 0.05 (ANOVA) and *P* < 0.05 (DMRT)

The effect of *H. indicus* aqueous extract on the microsomal protein concentration and cytochrome P-450-dependent monooxygenase enzyme is given in Table 3. The significantly (*F* > 0.05; *P* < 0.001) elevated levels of microsomal protein and monooxygenase enzymes: ethoxyresorufin-O-demethylase (EROD), pentoresorufin-O-demethylase (PROD), and p-nitrophenol hydroxylase (PNPH) in diabetic rats were restored to near-normal levels on treatment with *H. indicus* extract.

**Table 3:** Effect of *H. indicus* on liver microsomal protein and monooxigenase activity in normal and streptozotocin-induced diabetic rats

Groups	Dose (mg/kg bw/day)	Microsomal protein (mg/g organ wt)	Monooxygenase activity (nmol product/min/mg protein)		
			
			EROD	PROD	PNPH
Normal	35 ml/water	12.17 ± 0.98	0.22 ± 0.01	0.25 ± 0.02	0.95 ± 0.11
Diabetic control	35 ml/water	13.9 ± 0.75	0.42 ± 0.02	0.35 ± 0.03	1.00 ± 0.12
Diabetic + tolbutamide	100	12.9 ± 0.98*	0.22 ± 0.03*	0.25 ± 0.01*	0.96 ± 0.13*
Diabetic + *H. indicus*	500	13.1 ± 0.75*	0.24 ± 0.01*	0.24 ± 0.02*	0.91 ± 0.15*

EROD - ethoxyresorufin-O-demethylase; PROD - pentoresorufin-O-demethylase; PNPH - p-nitrophenol hydroxylase; Each value is mean ± SD for six rats in each group

* Values are statistically significantly when compared to diabetic control at *F* > 0.05 (ANOVA) and *P* < 0.05 (DMRT)

In diabetic rats there was a significant (*F* > 0.05; *P* < 0.001) increase in the serum electrolytes: sodium, potassium, and calcium. Oral administration of *H. indicus* aqueous extract significantly (*F* > 0.05; *P* < 0.001) reduced the levels of serum electrolytes when compared to normal rats [Table 4].

**Table 4:** Effect of *H. indicus* extract on serum electrolytes in normal and streptozotocin-induced diabetic rats

Groups	Dose (mg/kg bw/day)	Na^+^ (mEq/l)	K^+^ (mEq/l)	Ca^2+^ (mEq/l)
Normal	35 ml/water	145.00 ± 5.90	6.80 ± 1.25	7.38 ± 0.15
Diabetic control	35 ml/water	162.00 ± 2.36	7.90 ± 0.23	9.10 ± 0.23
Diabetic + tolbutamide	100	143.12 ± 2.12*	5.13 ± 0.62*	7.91 ± 0.56*
Diabetic + *H. indicus*	500	139.56 ± 4.23*	5.02 ± 0.54*	7.52 ± 0.63*

Each value is mean ± SD for six rats in each group

* Values are statistically significant when compared to diabetic control at *F* > 0.05 (ANOVA) and *P* < 0.05 (DMRT)

The effect of the *H. indicus* extract on the liver and kidney lipid peroxidation is given in Table 5. The significantly (*F* > 0.05; *P* < 0.001) elevated levels of thiobarbituric acid reactive substances (TBARS), tissue hydroperoxides, and tissue malondialdehyde in diabetic rats were reduced significantly (*F* > 0.05; *P* < 0.001) to near-normal levels upon treatment with *H. indicus* extract.

**Table 5:** Effect of *H. indicus* extract on liver and kidney TBARS, hydroperoxides, and malondialdehyde in normal and streptozotocin induced diabetic rats

Groups	Dose (mg/kg bw/day)	TBARS (mM/mg)		Hydroperoxides (nM/100 g tissue)		Tissue MDA (nmol/g wet weight)	
				
		Liver	Kidney	Liver	Kidney	Liver	Kidney
Normal	35 ml/water	0.68 ± 0.21	1.53 ± 1.08	70.64 ± 2.10	55.03 ± 2.09	295.4 ± 1.69	291.23 ± 1.02
Diabetic control	35 ml/water	1.45 ± 0.01	2.64 ± 0.36	115.57 ± 1.45	79.00 ± 1.01	365.3 ± 1.20	365.23 ± 1.65
Diabetic + tolbutamide	100	1.12 ± 1.01*	1.91 ± 0.27*	84.13 ± 1.32*	65.13 ± 1.11*	345.65 ± 1.02*	300.95 ± 1.24*
Diabetic + *H. indicus*	500	0.98 ± 1.01*	1.75 ± 0.08*	72.29 ± 0.13*	58.63 ± 1.30*	310.21 ± 1.54*	280.56 ± 1.36*

Each value is mean ± SD for six rats in each group

* Values are statistically significant when compared to diabetic control at *F* > 0.05 (ANOVA) and *P* < 0.05 (DMRT)

## Discussion

Our observations are in complete agreement with the reports by several workers that STZ-induced diabetes mellitus and insulin deficiency lead to increased blood glucose.[10] Administration of an aqueous extract of *H. indicus* roots (500 mg/kg bw/day) decreased the elevated blood glucose level within 5 h; prolonged administration might have stimulated the β-cells of islets of Langerhans to produce insulin. From the results it is assumed that the root extract could be responsible for stimulation of insulin release and the observed restoration of metabolic activities. Further, the observed blood glucose-lowering effect of the extract in STZ-induced diabetic rats could also possibly be due to increased peripheral glucose utilization. A number of other plants have also been shown to exert hypoglycemic activity through stimulation of insulin release.[11,12] The antihyperglycemic activity of the aqueous extract of *H. indicus* roots was comparable with tolbutamide, a standard hypoglycemic drug. Tolbutamide has long been used to treat diabetes and is known to act by stimulating insulin secretion through action on the pancreatic β-cells.

Insulin has been shown to be a potentiator of hexokinase and glucokinase.[13] The decreased levels of glycogen synthase, glucokinase, lactate dehydrogenase, succinate dehydrogenase and malate dehydrogenase may be due to decreased insulin levels in diabetic rats. Restoration of the concentration of glycolytic enzymes after oral administration of the aqueous extract might be due to an insulinomimetic action of *H. indicus* extract. Chemically (STZ)-induced diabetes has been shown to produce a partial or total deficiency of insulin that causes decrease in the concentration of glycolytic enzymes.[14]

Chemically induced diabetes has been shown to induce polymorphic alterations in the metabolic activities of cytochrome P-450-dependent monooxygenase enzyme systems.[15] In our study, the elevated concentrations of cytochrome P-450-dependent mono-oxygenase enzyme systems, such as EROD, PROD, and PNPH, may be due to hepatocellular damage caused by the formation of excessive oxygen free radicals. Oral administration of the aqueous extract restored the levels of hepatic drug-metabolizing enzymes.

Increased glucose oxidation in the presence of transition metals has been shown to produce membrane damage by membrane lipid peroxidation and protein glycation.[16] This could be the reason for the altered flux in electrolyte balance that resulted in the elevated extracellular concentration of sodium, potassium and calcium in STZ-induced diabetic rats. Administration of *H. indicus* aqueous extract reduced the LPO index and restored tissue antioxidants, and this could be the possible reason for the restoration of extracellular electrolyte concentrations.

Several studies have demonstrated the involvement of free radicals in the genesis of diabetes mellitus and their role in the induction of lipid peroxidation during diabetes.[17,18] It has been reported that in diabetes mellitus oxygen free radicals are generated by stimulating H_2_O_2_ *in vitro* as well as *in vivo* and in pancreatic β-cells.[19] In our study, the increased levels of TBARS, tissue malondialdehyde, and hydroperoxides in the liver and kidney of STZ-induced diabetic rats served as an index of elevated lipid peroxidation in the diabetic condition. The increase in lipid peroxidation indicates increased oxidative stress, generating more free radicals. Administration of *H. indicus* extract 500 mg/kg bw/day for the period of 12 weeks decreased the lipid peroxidation index. Significant reduction in lipid peroxidation can be attributed to the antioxidant activity of various phytochemicals present in the aqueous extract of the roots of *H. indicus*. Further, these results indicate the possibility that the major function of the extract may be in protecting vital tissues such as liver, kidney, pancreas, and brain, thereby reducing the complications of diabetes.

The oxidative stress in diabetes also includes shifts in redox balance resulting from altered carbohydrate and lipid metabolism, increased generation of reactive oxygen species (ROS) by glycation and lipid oxidation, and decreased antioxidant defenses. The cellular antioxidant scavenger system has been shown to be depleted under elevated oxidative stress–mediated tissue damage by a series of chemical reactions. Hyperglycemia-induced vascular damage has already been reported.[20]

It can be concluded from the results that the oral administration of an aqueous extract of *H. indicus* roots (500 mg/kg bw/day) is beneficial in controlling the blood glucose level and restores serum electrolytes, glucose metabolizing enzymes, and hepatic cytochrome P-450-dependent mono-oxygenase enzymes, and prevents lipid peroxidation-associated complications in STZ-induced experimental diabetic rats. Further pharmacological and biochemical investigations are underway to find out the active constituent responsible for the antidiabetic activity and to elucidate its mechanism of action.
